# A prediction model for the decline in renal function in people with type 2 diabetes mellitus: study protocol

**DOI:** 10.1186/s41512-021-00107-5

**Published:** 2021-11-18

**Authors:** Mariella Gregorich, Andreas Heinzel, Michael Kammer, Heike Meiselbach, Carsten Böger, Kai-Uwe Eckardt, Gert Mayer, Georg Heinze, Rainer Oberbauer

**Affiliations:** 1 Section for Clinical Biometrics, Center for Medical Statistics, Informatics and Intelligent Systems, Medical University of Vienna, Vienna, Austria; 2 Division of Nephrology and Dialysis, Department of Internal Medicine III, Medical University of Vienna, Vienna, Austria; 3 Department of Nephrology and Hypertension, Friedrich-Alexander Universität Erlangen-Nürnberg (FAU), Erlangen, Germany; 4 Department of Nephrology, University of Regensburg, University Hospital Regensburg, Regensburg, Germany; 5 Department of Nephrology and Medical Intensive Care, Charité Universitätsmedizin Berlin, Berlin, Germany; 6 Department of Internal Medicine IV (Nephrology and Hypertension), Medical University Innsbruck, Innsbruck, Austria

**Keywords:** Prediction modeling, Type 2 diabetes mellitus, Chronic kidney disease, Mixed model, Prognosis, Risk calculator

## Abstract

**Background:**

Chronic kidney disease (CKD) is a well-established complication in people with diabetes mellitus. Roughly one quarter of prevalent patients with diabetes exhibit a CKD stage of 3 or higher and the individual course of progression is highly variable. Therefore, there is a clear need to identify patients at high risk for fast progression and the implementation of preventative strategies. Existing prediction models of renal function decline, however, aim to assess the risk by artificially grouped patients prior to model building into risk strata defined by the categorization of the least-squares slope through the longitudinally fluctuating eGFR values, resulting in a loss of predictive precision and accuracy.

**Methods:**

This study protocol describes the development and validation of a prediction model for the longitudinal progression of renal function decline in Caucasian patients with type 2 diabetes mellitus (DM2). For development and internal-external validation, two prospective multicenter observational studies will be used (PROVALID and GCKD). The estimated glomerular filtration rate (eGFR) obtained at baseline and at all planned follow-up visits will be the longitudinal outcome. Demographics, clinical information and laboratory measurements available at a baseline visit will be used as predictors in addition to random country-specific intercepts to account for the clustered data. A multivariable mixed-effects model including the main effects of the clinical variables and their interactions with time will be fitted. In application, this model can be used to obtain personalized predictions of an eGFR trajectory conditional on baseline eGFR values. The final model will then undergo external validation using a third prospective cohort (DIACORE). The final prediction model will be made publicly available through the implementation of an *R shiny* web application.

**Discussion:**

Our proposed state-of-the-art methodology will be developed using multiple multicentre study cohorts of people with DM2 in various CKD stages at baseline, who have received modern therapeutic treatment strategies of diabetic kidney disease in contrast to previous models. Hence, we anticipate that the multivariable prediction model will aid as an additional informative tool to determine the patient-specific progression of renal function and provide a useful guide to early on identify individuals with DM2 at high risk for rapid progression.

## Introduction

Chronic kidney disease (CKD) has become an increasing global health problem, partly due to the continuously rising incidences of diabetes mellitus, obesity, and hypertension [1]. It is estimated that 8 to 16% of the world’s population suffer from CKD, and the rate is even higher in people with DM2, where cross-sectional studies report percentages of roughly 25% [2]. Ninety-five percent of people with early CKD are unaware of their disease [3]. However, early prediction of the continuous decline in kidney function could provide an additional resource for personalized preventative care [4]. Personalized risk assessment based on large study cohorts could therefore offer several potential benefits to the preventive care for people with DM2 such as early detection followed by monitoring to guide treatment and potentially slow the progression of the decline of kidney function as expressed by the estimated glomerular filtration rate (eGFR) [4]. Despite the accumulated knowledge with regard to CKD nowadays, identifying individuals at high risk for fast disease progression has proven to be challenging because longitudinal eGFR slopes not only vary between patients (inter-patient variability), but eGFR measurements also fluctuate within each patient over time (intra-patient variability) [5].

Nevertheless, several prediction models for the progression of renal function decline have previously been developed in various populations. It is of note that the models which were developed in studies from the last decade may no longer be applicable nowadays, because the new treatment classes of SGLT2-blocker and mineralocorticoid receptor antagonists have dramatically changed the course of CKD progression [6–8]. In addition, existing predictive models have mainly considered hard renal endpoints such as incidence or end-stage renal failure [9, 10]. Studies focused on renal function have mainly considered simplifications of the longitudinal eGFR trajectory by dichotomization of the expected eGFR slope. For instance, Subasi et al. [11] conducted a pilot study based on a randomized double-blinded treatment trial, the African American Study of Kidney Disease and Hypertension (AASK), in order to predict the rate of kidney function decline. The least-squares slopes of GFR decline from the 6-month time point until censoring were used to define rapid (i.e., an absolute slope < 3mL/min/1.73m^2^/year) and slow (i.e., absolute slope in between 1 and 3mL/min/1.73m^2^/year) progressors for this study. Similarly, Vigil et al. [12] aimed towards the identification of predictors of a rapid decline of renal function defined as an annual eGFR loss > 4 mL/min/1.73m^2^. However, covariates considered for selection in this analysis were chosen on the basis of their significance in univariable analysis (or by their clinical or biological relevance). Despite its popular use for data reduction, univariable prefiltering has been shown to lead to suboptimal or biased selection results [13]. Pena et al. [14] showed that a panel of novel biomarkers improves the prediction of renal function decline in people with DM2. In contrast to the other mentioned studies, renal function decline was not dichotomized. The eGFR slope was estimated using linear regression and then constituted the outcome of interest in its continuous form. People with an observed eGFR decline larger than −3mL/min/1.73m^2^ were considered stable, all others were considered to be rapid progressors in terms of kidney function decline. Then, a person’s risk was assessed by dichotomizing the observed decline in these two groups and comparing it with the predicted probabilities of eGFR decline. The threshold of −3mL/min/1.73m^2^ was selected based on literature, which however included older studies without the current state-of-the-art therapy of diabetic kidney disease, where a GFR loss of only 1mL/min//1.73m^2^/year is standard [14–17]. Furthermore, molecular data in regular clinical care is usually not easily available and no validated reference values exist for these markers.

In this study, we will apply state-of-the-art methodology to avoid deficiencies of previous attempts in prediction models for renal function decline. We will directly incorporate the repeated eGFR measurements per person as an outcome vector into a multivariable mixed-effects model accounting for the dependence of the repeated measurements of eGFR and the clustered data structure due to the use of two multicenter prospective study cohorts. We will also provide the methodology to condition a prediction on an available baseline value of eGFR, facilitating predictions regardless of whether a baseline eGFR is available or not. In contrast to the commonly applied approaches, the repeated values of eGFR across follow-up visits will not be used for slope estimation followed by categorization to infer groups of the severity of loss in kidney function (e.g., stable, mild, and rapid progression) prior to model development. The proposed approach will enable the identification of groups of patients with a high risk of rapid renal function decline by longitudinal prediction of subject-specific eGFR trajectories, thereby providing the probability of exceeding a prespecified cutpoint of eGFR decline per year. After the planned external validation, our model will be implemented as a web tool and can be clinically applied to identify people with DM2 at increased risk of rapid decline in kidney function, or as a supporting tool for clinicians in medical decision-making so that patients can be prompted to seek medical care before a significant deterioration in kidney function occurs.

### Objective

The main objective of the planned analysis is the development, internal-external, and external validation of a personalized prediction model for eGFR loss per year in Caucasian people with DM2. It is based on clinical information and laboratory measurements recorded at baseline. A secondary aim is the implementation of the externally validated prediction model as a publicly available web application to facilitate general applicability in clinical care based on data from recent eras using modern therapy.

## Research design and methods

### Data source

The study cohort for a model building comprises individuals from two distinct prospective observational studies (PROVALID, GCKD) covering a wide range of CKD states [18, 19]. For the purpose of external validation, a third prospective cohort study (DIACORE) will be used [20].

#### PROVALID - PROspective cohort study in patients with type 2 diabetes mellitus for VALIDation of biomarkers

The PROVALID study cohort consists of around 4000 people with DM2 of whom information on medical history, physical status, laboratory measurements, and medication have been prospectively collected. Patients who are being taken care of at the primary healthcare level in five European countries (Austria, Hungary, Netherlands, Poland, and Scotland) were recruited between 2011 and 2015 and followed for at least 5 years. Participants had to be aged 18–75 years and had an incident or prevalent DM2 defined as treatment with hypoglycaemic drugs or according to ADA guidelines at the time point of study entry. The presence of CKD at study entry was not an exclusion or inclusion criterion. For the full inclusion and exclusion criteria of patients, see Table 1. The aim of the study was to investigate regional differences in the course of diabetic nephropathy to determine the 5-year cumulative incidence of renal and cardiovascular outcomes and to identify predictive biomarkers for the eGFR trajectory in patients with DM2.

**Table 1:** Overview of the inclusion and exclusion criteria of PROVALID, GCKD, and DIACORE

Study cohort	Study setting	
	Inclusion	Exclusion
PROVALID	▪ Aged 18–75 years ▪ Incident or prevalent DM2 with/without CKD	• Active malignancy requiring chemotherapy
GCKD	▪ Aged 18–74 years ▪ Caucasian ethnicity ▪ eGFR: 30–60 mL/min/1.73m^2^with or without urinary albumin excretion ▪ eGFR > 60 mL/min/1.73m^2^ - Albumin excretion of > 300mg/g creatinine - Proteinuria > 500mg/day	▪ Active malignancy in the 24 months prior to inclusion ▪ Renal or other transplantations ▪ NYHA IV heart failure
DIACORE	▪ Aged ≥ 18 years ▪ Caucasian ethnicity ▪ Prevalent DM2	▪ On chronic renal replacement therapy ▪ Active malignancy ▪ Pregnancy ▪ Further diseases and infections

#### GCKD—German chronic kidney disease

The prospective cohort study GCKD consists of around 5000 people with CKD. Patients aged 18–74 years and suffering from CKD were included in case of an eGFR between 30 and 60 ml/min/1.73m^2^ with or without urinary albumin excretion (CKD KDIGO Stage 3) or a better preserved eGFR in the presence of urinary albumin excretion >300 or protein excretion >500mg/day, and were followed up to 10 years. For the full inclusion and exclusion criteria of patients, see Table 1. Out of the 5000 recruited individuals, 1800 had diabetes and therefore represent the relevant patient subset for the prediction model in this work. The follow-up visits were set up in bi-yearly intervals. The main objective of the GCKD study cohort was to identify and validate risk factors for CKD, end-stage renal disease (ESRD), and cardiovascular disease (CVD) events.

#### DIACORE—the DIAbetes COhoRtE study

DIACORE is a prospective observational cohort study consisting of 6000 people with prevalent DM2 in Germany with at least 10 years of follow-up. The main objective of this study was the investigation of risk factors associated with the development and progression of diabetic complications through biosampling using high-throughput technologies for the collected biosamples (i.e., transcriptomics, proteomics, and metabolomics). To this end, patient information and blood samples were taken at baseline and at every bi-yearly follow-up visit for at least 10 years after study initiation.

### Target population

The following inclusion criteria will be applied to the individuals from the PROVALID, GCKD, and DIACORE studies for the development and validation of the prediction model in this work:

- Caucasian ethnicity
- Presence of DM2 at baseline
- eGFR > 30 mL/min/1.73m^2^ at baseline
- Aged 18–75 years at baseline
- At least three visits with recorded serum creatinine measurements (including baseline)
- At least 2 years of follow-up

### Study outcome

The primary outcome of interest for the study is the annual decline in renal function, derived through the use of at least 3 follow-up measurements of eGFR. The eGFR values will be calculated by the equation of the Chronic Kidney Disease Epidemiology Collaboration (CKD-EPI) using the patient’s race, sex, age, and serum creatinine level [21].

### Clinical predictor variables

The candidate variables for consideration in the prediction model were selected by medical experts. Variables of general clinical availability, ease in acquisition, and with clinical acceptance for inclusion in the model will be prioritized. Hence, the following variables will be included in the multivariable outcome model for the decline in renal function (see Table 2): age, sex, body mass index (BMI), smoking status (ever/never smoked), HbA_1c_, urine albumin-to-creatinine ratio (UACR), presence of glucose-lowering medication, presence of lipid-lowering medication, presence of blood pressure-lowering medication, systolic and diastolic blood pressure, hemoglobin, and serum cholesterol. All predictors were measured at baseline, i.e., the first visit after the patient was included in either of the study cohorts. Due to the varying depth of information regarding the medication of an individual in the three cohorts, only drug indication classes will be included as a binary entry (y/n).

**Table 2:** Clinical baseline information of study participants within PROVALID, GCKD, and DIACORE

Variable type	Variable	Level of measurement	Unit
Demographics	Age	Continuous	Years
	Sex	Binary	Female-male
Physical status	Smoking	Binary	Never-ever
	BMI	Continuous	kg/m^2^
Laboratory measurements	eGFR	Continuous	mL/min/1.73m^2^
	Systolic blood pressure	Continuous	mmHg
	Diastolic blood pressure	Continuous	mmHg
	HbA_1c_	Continuous	mmol/mol
	Serum cholesterol	Continuous	mg/dl
	Hemoglobin	Continuous	*μ*mol/L
	UACR	Continuous	mg/g
Intake of medication	Blood pressure-lowering	Binary	Yes-no
	Glucose-lowering	Binary	Yes-no
	Lipid-lowering	Binary	Yes-no

### Sample size

Overall, 13 predictors will be included in the prediction model. Guidelines regarding the minimum required sample size for the development of a new multivariable prediction model have been recently proposed by Riley et al. [22]. Using the accompanying R package “pmsampsize” with an assumed average value for eGFR of 78.4mL/min/1.73m^2^ and a standard deviation of 21.4 mL/min/1.73m^2^ [23] in the population, an anticipated *R*^2^ of the model of 0.3 [24] and a shrinkage factor of 0.9, the minimum sample size aimed at minimization of overfit and the precise estimation of the parameters of a prediction model with 13 predictors is 271. Further, since regularization is not taken into consideration in model development due to the abundance of data relative to the number of predictors, the computation with a shrinkage parameter of 0.99 results in a sample size of 3048 required subjects. However, a much larger *R*^2^ is expected, which is why a minimum sample size of 1569 is required for a *R*^2^ of 0.5 and 903 participants for 0.7, respectively. Nevertheless, the development cohort will comprise around 5800 subjects and thus will exceed the minimum required sample size by far.

### Statistical analysis

Patient’s baseline characteristics will be described for the study cohorts separately, using mean and standard deviation for continuous variables, or median and interquartile range in case of non-normality, and absolute and relative frequencies for categorical variables. Skewed variables will be transformed by the logarithm before the analysis. Data availability and the fraction of missingness will be assessed and reported for each variable. As the fraction of missing data is expected to be low, a complete case analysis will be carried out. Data screening through the computation of sample characteristics and visual inspections to identify the presence of missing values, skewed variable distributions, and outliers, as well as reporting of conducted screening activities will adhere to the conceptual framework for initial data analysis by Huebner et al. [25]. All analysis will be performed with the software R version 4.0.2.

### Prediction model development

People adhering to the inclusion and exclusion criteria from either the PROVALID or GCKD study will be considered for model building. Mixed-effects modeling accounts for the dependencies between repeated measurements per person over time and the similarity among people belonging to the same study cohort, hence they are commonly employed to analyze longitudinal and clustered data. Here, we will consider hierarchically nested random effects for the country and people within a country, such that the heterogeneity of eGFR trajectories between the five countries within the development cohort can be modeled appropriately [26]. More specifically, we will use random effects for the intercept and the slope of the repeated eGFR measurements over time and an additional random intercept for the country. We will include the baseline eGFR value in the outcome vector to provide more stable estimates of variability. When applying the model, any available eGFR measurements of an individual for whom eGFR trajectory predictions should be obtained can be used to improve predictions. A similar approach has been conducted by Selles et al. [27] to develop a prediction model not only able to deal with multiple measurements per subject but also able to allow these repeated clinical measurements to contribute to the prediction of an unseen individual. The set of clinical variables outlined in Table 2 will constitute the fixed effects. Automated variable selection will not be conducted as the set of predictors was chosen by background knowledge. Furthermore, due to the large sample size available for estimation overfitting is expected to be minimal, such that further regularization of the predictor effects will be omitted. Clinically relevant pairwise interactions of the independent variables with time will be investigated and added if their inclusion improves the prediction accuracy of the model as determined by the Akaike information criterion (AIC). Any model misspecification, e.g., regarding functional forms of the variables in the model, will be assessed using a plot of the marginal residuals versus the individual variables. Variables with non-linear relationships with the outcome as exhibited by these residual plots will then be modeled using restricted cubic splines to improve the model fit [28]. In addition, the relevance of the inclusion of such non-linear terms for improving the model fit will be evaluated in terms of the AIC.

More formally, the model for the continuous eGFR values *Y*_*it*_ at time point *t* (*t* = 0, …, *T*) for subject *i* (*i* = 1, …, *n*) is defined by

1 $$ {\mathrm{Y}}_{\mathrm{i}\mathrm{t}}={\upbeta}_0+{\mathrm{a}}_{\mathrm{i}}+{\upbeta}_1X+{\upbeta}_2\mathrm{t}+{\upbeta}_3\mathrm{tX}+{\mathrm{b}}_{0\mathrm{i}}+{\mathrm{b}}_{1\mathrm{i}}\mathrm{t}+{\upepsilon}_{\mathrm{i}\mathrm{t}}, $$

with normally distributed residuals ${\epsilon}_{it}\sim N\left(0,{\sigma}_{\epsilon}^2\right)$ with mean 0 and variance ${\sigma}_{\epsilon}^2$, random country-specific intercepts assumed to be *a*_*i*_~*N*(0, *τ*^2^), the random coefficients (*b*_0*i*_, *b*_1*i*_)~*MN*(0, *G*) (where MN denotes the bivariate normal distribution with an unspecified covariance matrix G, defined by the diagonal elements ${\upsigma}_0^2$ and ${\upsigma}_1^2$ and the off-diagonal σ_01_) and the vector of regression coefficients for the clinical independent variables *β* = (*β*_0_, *β*_1_, *β*_2_, *β*_3_). It should be noted that for simplicity of description, we here consider a single clinical predictor variable *X*, where *β*_1_ and *β*_3_ represent the regression coefficients corresponding to the clinical predictor variable and its interaction with time. Without loss of generality, more clinical predictors can be added, each with two regression coefficients for the baseline effects of the predictor and the corresponding interaction with time. When considering restricted cubic splines for a variable, this variable will be represented by several corresponding basis functions in (1).

Model (1) allows to prognosticate baseline and follow-up values of eGFR for a new person *j* given the value of the clinical predictor *X*, assuming that for that new person the random intercept and slope are equal to their expected values of 0. Further, we will assume the expected random country effect of *a*_*j*_ = 0 for the prediction of eGFR in a new individual. In order to incorporate a baseline eGFR measurement *y*_*j*0_ into the prediction, a best linear unbiased predictor (BLUP) of the individual’s random effects can be obtained through the posterior distribution of the random effects. The random intercept for that new subject *j* is then estimated by

2 $$ {\hat{\mathrm{b}}}_{0\mathrm{j}}=\frac{\upsigma_0^2\left({\mathrm{y}}_{\mathrm{j}0}-{\hat{\mathrm{y}}}_{\mathrm{j}0}\right)}{\upsigma_0^2+{\upsigma}_{\upvarepsilon}^2}, $$

where ${\hat{\mathrm{y}}}_{\mathrm{j}0}$ denotes the estimated baseline value from the fixed effects, ${\sigma}_{\epsilon}^2$ denotes the variance of the random error and ${\sigma}_0^2$ the variance of the random intercept in the covariance matrix of the random coefficients *G*. The estimated random slope ${\hat{b}}_{1j}$ for the new individual can be obtained by

3 $$ {\hat{\mathrm{b}}}_{1\mathrm{j}}=\frac{\upsigma_{01}{\hat{\mathrm{b}}}_{0\mathrm{j}}}{\upsigma_0^2} $$

In actual predictions, estimates are plugged in for ${\sigma}_o^2$, σ_01_, and ${\upsigma}_{\upvarepsilon}^2$. The preliminary prediction ${\hat{y}}_{jt}$ for the new subject *j* at time *t* can then be updated by

4 $$ {\overset{\sim }{\mathrm{y}}}_{\mathrm{jt}}={\hat{\mathrm{y}}}_{\mathrm{jt}}+{\hat{\mathrm{b}}}_{0\mathrm{j}}+\mathrm{t}{\hat{\mathrm{b}}}_{1\mathrm{j}} $$

The predicted eGFR slope for a new individual, combining fixed and random effects, is finally given by $d\tilde{y}_{j}:= d{\overset{\sim }{\mathrm{y}}}_{\mathrm{j}\mathrm{t}}/ dt={\hat{\upbeta}}_2+{\hat{\upbeta}}_3{\mathrm{X}}_{\mathrm{j}}+{\hat{\mathrm{b}}}_{1\mathrm{j}}$ with ${\overset{\sim }{V}}_j$ expressing the variance of the predicted eGFR slope of individual *j*.

Given a correct model specification, the probability of progression can then be obtained by a normal approximation:

5 $$ \Pr \left(\mathrm{Z}={\mathrm{z}}_{\mathrm{j}}\right)=1-\Phi \left(\frac{{\mathrm{C}}_{\mathrm{p}}-d{\overset{\sim }{\mathrm{y}}}_{\mathrm{j}}}{\sqrt{{\overset{\sim }{V}}_j}}\right) $$

where *z*_*j*_, *j* ∈ {1, 2} denotes the membership of belonging to one of the two progression groups: rapid and stable progression, and Φ(x) the standard normal distribution function at *x*. *C*_*p*_ is the corresponding subject-specific cutpoint for defining progression of kidney decline, which will be set to −3mL/min/1.73m^2^/year at default or can be specified by the intended user in the web application (see Section "Web implementation") [14–16]. Now, one can compute the expected probability of belonging to either of the eGFR progression groups given a patient’s baseline characteristics and the baseline eGFR.

For all statistical analyses, the freely available software R will be used. Mainly, the packages *nlme* and *JMbayes* will be considered for the implementation of the linear mixed model and the subject-specific prediction informed by the baseline eGFR measurement. The function *lme()* from the package *nlme* will be used to fit the linear mixed model and the function *IndvPred_lme()* from the package *JMbayes* to obtain the updated predictions and 95% prediction intervals for a new subject.

### Model evaluation, performance, and external validation

An analysis of the conditional residuals will be conducted to check correct model specification, e.g., normality, linearity, and homoscedasticity of the error terms. Next, the importance of each independent variable will also be evaluated by the drop in the adjusted *R*^2^, i.e., the loss in estimated explained variation when the respective predictor is removed from the model.

The performance of the prediction model will be evaluated by an internal-external validation procedure to obtain a reliable assessment of its generalizability of prediction to unseen data [29]. The unit for the non-random data splitting will be the six countries within the PROVALID and GCKD studies (GCKD: Germany; PROVALID: Austria, Hungary, Netherlands, Poland, and Scotland) such that for each iteration, one regional group with its study participants are withheld from model training and then used for testing.

The prediction performance will further be assessed through:

- Discrimination in terms of Kendall-tau-b concordance correlation coefficients at each time point, i.e., the concordance of the predicted eGFR values at time *t* obtained by Eq. (4) and the actual observed eGFR in each pair of patients
- Calibration in terms of the slope of the calibration curve by
  I. Regressing the predicted eGFR on the actual eGFR values at each time point
  II. Plotting the estimated mean risk for fast progression per decile against the observed ratio of fast progression within each risk decile
- Explained variation in terms of adjusted *R*^2^, i.e., the proportion of explained variability in the outcome by the independent variables

The external validity of the model will be examined by applying the final prediction model to the DIACORE study cohort and evaluating the three performance measures outlined above.

### Model presentation

Reporting of the model development and the final prediction model will adhere to the Transparent Reporting of a Multivariable Prediction Model for Individual Prognosis or Diagnosis (TRIPOD) statement, a checklist of items that ought to be reported when publishing a statistical prediction model [30]. The results of this analysis will be published in a peer-review journal. In particular, the final regression model will be presented using regression coefficients and 95% confidence intervals. Further, model reporting will include the covariance matrix of the random effects, the error variance, and the regression formula to allow independent application and validation of the model. Visualizations of results will be generated to improve model literacy for non-statistical readers and users of the web-based calculator.

### Web implementation

The externally validated model will be implemented as an interactive prediction calculator, made available online as a web application to support clinical care and medical decision-making with regard to prevention and treatment of chronic kidney function decline in people with DM2. The interactive web application will be created with the help of the R development framework *shiny* to allow for individual input and the computation of patient-specific predictions given their baseline characteristics. The web application will offer two user interfaces to choose from:

1. A patient interface with a web layout consisting of limited input controls (i.e., text fields or checkboxes to enter age, sex, eGFR, and UACR)
2. A clinician interface with a layout featuring input controls for all predictors (i.e., includes laboratory measurements, medication) within the validated prediction model

Both interfaces will be based on the same prediction model formula, with predictors that are excluded from the web layout in the patient interface being fixed as the average value for the continuous variables and the value with the highest relative frequency for the categorical variables. As a result, the use of the model should not only be made available to clinical staff but also to patients themselves.

The output of the web application will contain the following components:

- The eGFR value estimated by the model in 1–5 years with 95% model-based prediction intervals
- The predicted eGFR decline per year with 95% model-based prediction interval
- The proportion of people with higher predicted eGFR loss
- The relative risk, i.e., the probability for a rapid eGFR decline for the predicted interval divided by the probability of a rapid eGFR decline for a person with equal age, sex, and baseline eGFR, but most favorable values of all other clinical baseline variables (with rapid decline specified by the intended user)
- A visual representation of the results:
- The visual communication of the results to the user will illustrate the estimated eGFR decline in the context of the distribution of predicted eGFR declines in the development cohort. In addition, a figure will be generated that shows the predicted trajectory of eGFR over the next 5 years with a 95% prediction interval.

A further option will be the possibility of risk assessment of rapid and stable eGFR decline. However, the choice of cutpoint selection for the categorization will be left to the user of the web-based implementation, in that the user can freely enter a suitable cutpoint for the eGFR classification into rapid and stable progression, whereby the risk of the new individual is estimated. By default, the eGFR cutpoint will be set to −3ml/min/1.73m^2^.

## Discussion

In this study, we have described the protocol for the planned development and validation of a new clinical prediction model for the decline of renal function in Caucasian people with DM2 to aid decision-making in clinical care. Our methodological approach will be based on a multivariable linear mixed model able to account for the dependence of clinical parameters per subject over time and for the similarity of individuals within the same country. Previous studies have employed risk prediction based on (multinomial) logistic regression models for which as a dependent variable the dichotomized eGFR slope was used, after estimation by least squares using the repeated measurements and then partitioning at arbitrary cutpoints. Each eGFR strata then covers different intervals on the eGFR slope spectrum and is supposedly indicative of varying severity of the progression of renal function loss. However, this approach suffers from several shortcomings. First, individual eGFR slopes can only be estimated very inaccurately, as only very few data points at non-equidistant time points are available for each patient [5]. Moreover, the categorization of the resulting eGFR slopes adds another layer of possible bias as it leads to subjects close on the continuous spectrum but on the opposite sides of the cutpoint being characterized as different [31]. By contrast, our approach avoids the issues associated with the imprecise estimation of slopes and inappropriate dichotomization prior to model development and takes the observed data fully into consideration when estimating the parameters of the model. In addition, we include the baseline measurements of eGFR into the outcome vector of an individual, as it is subject to the same measurement error as later eGFR measurements. In this way, these baseline measurements contribute to a more precise estimation of the error variance. Nevertheless, available eGFR measurements at the time of prediction (application of the model) can still be incorporated in our approach to optimize the predicted eGFR trajectory. In addition, the clustered data structure due to the two multicentre cohorts (GCKD and PROVALID) for model training will be taken into account by including an additional random intercept for the country in contrast to existing models. Therefore, the potential heterogeneity of model performance across the countries, which are distinct between GCKD and PROVALID, is prevented and hence, the generalizability to unseen individuals is improved.

However, this study will have a few limitations. First, it is to be expected that for some individuals not all creatinine measurements will be available, in particular, at later time points. Such random missingness is common in prospective longitudinal studies. Even in the case of informative missingness, by capturing much information about the patients by means of domain-expertise-selected clinical baseline variables, which are included in multivariable modeling, the mixed model is still able to provide unbiased predictions. Second, we will not include extensive biosampling data (e.g., metabolomics, proteomics, lipidomics) in the model. The inclusion of these biomarkers may improve the predictive accuracy of the model, but the scientific evidence for an added value in prediction is still scarce and the biomarkers have no validated test characteristics. We will rather prioritize clinical variables that are regularly collected and which are widely available to ensure the broad applicability of our model. Lastly, the lack of standardization of creatinine assays across cohorts inflicts variability of the clinical laboratory serum creatinine measurements and hence can induce potential bias in calculating eGFR.

The key strength of this study includes not only the refined methodology in the development of the prediction model, but also its large sample size due to the usage of two prospective observational cohort studies PROVALID and GCKD for model development. This ensures stable parameter estimation of the model. Another strength is the availability of an additional, independent prospective cohort study, DIACORE, for external validation after model development. To our knowledge, this is the first prediction model specifically developed for a central European population suffering from DM2 and covering a wide range of CKD stages.

Overall, we have outlined a robustly developed and validated clinical prediction model which will generalise to a wide range of patients with regard to initial CKD progression suffering from DM2. It will also be derived from a diverse and multinational population using two large studies and will be used to predict deterioration in renal function in DM2 patients to improve further eGFR development through early intervention in patients at high risk for the rapid progression of renal decline.

## Conclusion

This will be a prediction model for eGFR loss in Caucasian subjects with DM2 that will use data from recent observational multinational studies. By adhering to transparent reporting procedures and current best practice for model development and validation, we will minimize the risk of bias when our prediction model is applied in the context of primary prevention of progression of CKD in people with DM2.

## Data Availability

Not applicable.
